# Using acetone for rapid PCR‐amplifiable DNA extraction from recalcitrant woody plant taxa

**DOI:** 10.1002/aps3.11403

**Published:** 2020-12-03

**Authors:** Fred E. Gouker, Yonghong Guo, Margaret R. Pooler

**Affiliations:** ^1^ U.S. Department of Agriculture Agricultural Research Service U.S. National Arboretum, Floral and Nursery Plants Research Unit Beltsville Maryland USA

**Keywords:** DNA extraction, inhibitors, real‐time PCR, woody plants

## Abstract

**Premise:**

Quick and effective DNA extraction from plants for subsequent PCR amplification is sometimes challenging when working across diverse plant taxa that may contain a variety of inhibitory compounds. Time‐consuming methods may be needed to overcome these inhibitory effects as well as the effects of various preservation and collection methods to extract DNA from leaf samples. Our objective was to develop a rapid DNA extraction protocol that could be used with diverse plant taxa to produce high‐quality DNA suitable for downstream PCR applications.

**Methods and Results:**

We tested the efficacy of acetone in extracting DNA from fresh, frozen, oven‐dried, acetone‐fixed, and herbarium leaf material of 22 species from 16 woody and herbaceous plant families. An improved simplified DNA extraction protocol was developed using acetone‐fixed leaf material. The addition of 1% sodium dodecyl sulfate solution resulted in the optimal extraction from all tissue samples. The DNA resulting from the extraction protocol was readily amplified using real‐time PCR assays.

**Conclusions:**

The protocol described here resulted in the extraction of DNA from recalcitrant plant species that was of sufficient quality and quantity for PCR amplification, as indicated by the low threshold cycle values from real‐time assays. This method is simple, fast, and cost‐effective, and is a reliable tool for extracting high‐quality DNA from plant material containing PCR inhibitors.

The amplification of DNA from plants can be inhibited by compounds in plant tissues, including polysaccharides, phenolics, and terpenoids, that are difficult to eliminate with routine DNA extraction protocols (Jobes et al., 1995; Sharma et al., 2002; Kontanis and Reed, 2006). Such compounds are common in woody plant species, where DNA quality is often further compromised by the dehydration, lyophilization, or desiccation of samples prior to extraction. Many modified protocols and commercial kits developed specifically for plant DNA extraction offer improvements but can still result in failed or poor amplification (Fang et al., 1992; Dilworth and Frey, 2000; Sharma et al., 2002; Samarakoon et al., 2013; Siegel et al., 2017).

Research labs, botanic gardens, and arboreta that work in woody plant breeding, conservation, and population genetics often extract DNA from hundreds of samples, which requires a simple DNA extraction protocol that is effective in diverse taxa regardless of the sample collection and storage conditions used (e.g., fresh, frozen, freeze‐dried, or herbarium tissue). To develop such a protocol, we extracted DNA from 22 taxa in 16 plant families using only three chemical reagents (acetone, 1% sodium dodecyl sulfate [SDS], and 1× Tris–EDTA [TE]) that have been used in previously published methods (Samarakoon et al., 2013; Barbier et al., 2019) but not in combination at the concentrations we tested. This study tested multiple isolation techniques and evaluated the quality of the resulting DNA using real‐time PCR assays.

## METHODS AND RESULTS

### Plant material

The plant materials initially tested were newly expanded leaves from wild‐collected *Sassafras albidum* (Nutt.) Nees, which is recalcitrant to DNA extraction using commercial kits. Upon collection, the leaf material was preserved by either flash‐freezing in liquid nitrogen and storing at −80°C, oven‐drying at 37°C, or fixing in 100% acetone (Table 1). The extracted DNA was tested for its amplification efficiency using real‐time PCR. Based on the favorable results from the simple acetone‐based DNA extraction in *S. albidum*, plant samples from 12 diverse families (Aquifoliaceae, Buxaceae, Fabaceae, Fagaceae, Lauraceae, Oleaceae, Orchidaceae, Pinaceae, Poaceae, Rosaceae, Sapindaceae, and Solanaceae; Table 2) were subsequently tested using freshly collected leaves fixed in acetone. Eight additional plant species were tested using herbarium specimens (Appendix 1) from the U.S. National Arboretum (Table 2). These taxa were selected based on their diversity, ensuring a representation of woody and herbaceous material, and the availability of material for sampling.

**Table 1:** DNA quantity and the results of real‐time PCR amplification using the indicated primers from fresh, dried, and acetone‐fixed *Sassafras albidum* leaf tissue.

Leaf material	DNA yield (ng)^a^	C_t_ ^a^, ^b^	
		ITS‐p3/u4	SAFG‐57
Fresh	269 ± 36	28.61 ± 0.44	26.94 ± 0.13
Dry	432 ± 82	28.34 ± 0.31	26.62 ± 0.01
Acetone‐fixed	153 ± 65	29.27 ± 0.12	27.91± 0.52

Note

C_t_ = threshold cycle; ITS‐p3/u4 = internal transcribed spacer (ITS) of nuclear ribosomal DNA plant universal primer pair; SAFG‐57 = *Sassafras albidum* transcriptome‐derived simple sequence repeat primer pair.

^a^ *n* = 3, mean ± SD.

^b^ Using 2 ng DNA per reaction.

**Table 2:** DNA yield and real‐time PCR threshold cycle (C_t_) values from acetone‐fixed leaf disks from fresh samples of 14 plant taxa and herbarium samples of eight plant taxa.

Family^a^	Species	DNA yield (ng)	C_t_ value^b^
Fresh samples			
Aquifoliaceae	*Ilex cornuta*	664	21.37 ± 0.06
Buxaceae	*Buxus sempervirens*	115	26.69 ± 0.17
Fabaceae	*Cladrastis kentukea*	11	26.21 ± 0.03
Fabaceae	*Cercis glabra* (control)^c^	80	21.72 ± 0.04
Fagaceae	*Quercus bicolor*	59	24.81 ± 0.07
Lauraceae	*Sassafras albidum*	124	30.96 ± 0.15
Oleaceae	*Jasminum nitidum*	115	19.08 ± 0.02
Orchidaceae	*Phalaenopsis* sp.	532	31.75 ± 0.04
Pinaceae	*Tsuga chinensis*	308	23.41 ± 0.05
Pinaceae	*Picea orientalis*	316	20.56 ± 0.03
Poaceae	*Agrostis capillaris*	24	28.67 ± 0.27
Rosaceae	*Prunus serrulata*	1080	16.05 ± 0.08
Sapindaceae	*Acer saccharum*	53	24.56 ± 0.05
Solanaceae	*Nicotiana edwardsonii*	181	21.04 ± 0.03
Water	(negative control)	0	>40
Herbarium samples			
Aquifoliaceae	*Ilex opaca*	632	>40
Dryopteridaceae	*Onoclea sensibilis*	131	>40
Hemerocallidaceae	*Hemerocallis thunbergii*	102	>40
Phrymaceae	*Mimulus aurantiacus*	180	>40
Pinaceae	*Pinus pinceana*	550	30.61 ± 0.85
Poaceae	*Panicum virgatum*	171	26.41 ± 0.06
Salicaceae	*Salix caroliniana*	238	27.01 ± 0.01
Solanaceae	*Solanum oplocense*	74	29.70 ± 0.24

^a^ Results using primer pair ITS‐p3/u4 and 1 ng of DNA are shown for the fresh samples, and results using primer pair ITS‐3/4 and 2 ng of DNA are shown for herbarium samples.

^b^ *n* = 3, mean ± SD for C_t_ values.

^c^ This species was used as the positive control for the primers ITS‐p3/u4, following the methods of Cheng et al. (2016).

### DNA extraction and quantification

A detailed protocol for DNA extraction and quantification is presented in Appendix 2. A clean cork borer sterilized with 95% ethanol was used to take three disks (5 mm in diameter, 5–10 mg each) of leaf material. The leaf disks were placed into a 1.5‐mL microcentrifuge tube containing 0.5–1.0 mL of 100% acetone (enough to fully immerse the leaf tissue) at room temperature. The acetone was changed until the leaves turned white, after which the samples were stored at room temperature in the acetone until the DNA extraction. A single acetone‐fixed leaf disk was air‐dried, ground into small pieces using a metal rod in a 1.5‐mL centrifuge tube, and incubated in buffer (200 µL of 1× TE with 1% SDS) at 90°C for 20 min. The tubes were vortexed twice during the incubation and then chilled on ice for 5 min. The tubes were centrifuged at 14,000 rpm for 5 min, after which the supernatant was transferred to a new 1.5‐mL tube. An equal volume of 100% acetone was added and mixed by vortexing, and the mixture was then centrifuged at 6000 rpm for 1 min. The supernatant was discarded, and the resulting pellet was resuspended in 100 µL of nuclease‐free water. Three samples from each DNA extraction were quantified using the Qubit dsDNA HS Assay kit and the Qubit 3.0 fluorometer (Thermo Fisher Scientific, Waltham, Massachusetts, USA), following the manufacturer’s instructions. The average Qubit‐based concentration was used to calculate the total amount of DNA extracted from the initial sample.

### Real‐time PCR amplification

We used universal plant PCR primers to determine whether the DNA extracted using these protocols was suitable for downstream PCR amplification. These included the internal transcribed spacer (ITS) of nuclear ribosomal DNA plant universal primer pair ITS‐p3/u4 (5′‐YGACTCTCGGCAACGGATA‐3′, 5′‐RGTTTCTTTTCCTCCGCTTA‐3′) (Cheng et al., 2016) for all taxa; the ITS‐3/4 primer pair (5′‐GCATCGATGAAGAACGCAGC‐3′, 5′‐TCCTCCGCTTAATTGATATGC‐3′ (White et al., 1990) for herbarium samples; and a *S. albidum* transcriptome‐derived simple sequence repeat primer pair, SAFG‐57 (5′‐AGTCCCTCTCCCTCAACAATATG‐3′, 5′‐GGAGGGTTTGGTTTTGGATG‐3′) (F. E. Gouker, unpublished data), for the validation of a species‐specific primer pair. All primers were manufactured by Integrated DNA Technologies (Coralville, Iowa, USA).

Real‐time PCR assays were performed as three replicates per sample in a 10‐µL final reaction volume using a Bio‐Rad CFX96 Touch Real‐Time System C1000 Thermal Cycler (Bio‐Rad Laboratories, Hercules, California, USA). The PCR mixture contained 0.5 µL of each 10‐µM primer, 5.0 µL of Precision Melt Supermix (Bio‐Rad Laboratories), 2.0 µL of nuclease‐free water, and 1–2 ng of template DNA. The PCR profile consisted of 5 min of preheating at 95°C, followed by 40 cycles of 10 s of denaturation at 95°C, 10 s of annealing at 52°C, and 10 s of extension at 72°C. The plant universal primer pair ITS‐p3/u4 was used to test the amplification of DNA from all plant samples, and ITS‐3/4 was also used to test the amplification of DNA from herbarium specimens.

### DNA quality and quantity

Experiments using *S. albidum* indicated that oven‐dried samples resulted in the highest DNA yield (432 ng), which was 1.6 and 2.8 times greater than the yield from fresh and fixed leaf disk samples, respectively (Table 1). Quantification with real‐time PCR showed similar threshold cycle (C_t_) values from all three sample preparation methods (Table 1). When tested on 13 additional plant taxa, the acetone and SDS extraction method from fresh tissue resulted in DNA yields of 11 ng to 1080 ng, with successful PCR amplification from all samples tested (Table 2). The real‐time PCR assays were performed with 1–2 ng of template DNA for each sample, and all species had C_t_ values <35, indicating a reliable amplification efficiency. Extraction with acetone and SDS also resulted in amplifiable DNA from the recalcitrant woody plant species (*S. albidum*, *Tsuga chinensis* (Franch.) E. Pritz. in Diels, *Picea orientalis* (L.) Peterm., *Prunus serrulata* Lindl., *Acer saccharum* Marshall). The eight herbarium specimens yielded DNA using the acetone and SDS method, but the DNA was not consistently amplified using the primer pair ITS‐p3/u4 (data not shown). Amplification using the primer pair ITS‐3/4 was more consistent, but from only four of the eight samples (*Pinus pinceana* Gordon, *Panicum virgatum* L., *Salix caroliniana* Michx., and *Solanum oplocense* Hawkes) (Table 2). The herbarium samples that yielded no amplification product likely had significantly degraded DNA.

## CONCLUSIONS

We developed a simplified and quick DNA extraction method for herbaceous, woody, and herbarium plant specimens that uses only three chemical reagents (TE buffer, acetone, and SDS) and a single 5‐mm‐diameter leaf disk per sample without additional alcohol precipitation. The quality and quantity of the resulting DNA are sufficient for real‐time PCR amplification and quantification of most samples, with the exception of some herbarium specimens.

Other recently reported methods have used similar modifications of existing protocols to optimize nucleic acid extraction from recalcitrant plants, but those methods rely on the modification of protocols developed for purchased extraction kits (Samarakoon et al., 2013) or use additional chemicals such as cetyltrimethylammonium bromide (CTAB), phenol, or chloroform for extraction, or salts and alcohol for multiple precipitation steps post‐extraction (Samarakoon et al., 2013; Siegel et al., 2017; Barbier et al., 2019). The methods described here demonstrate the efficacy of tissue preservation and subsequent extraction in acetone with only the addition of 1% SDS, and without additional alcohol precipitation steps. This method provides a low‐cost and rapid alternative to extract DNA from fresh, frozen, oven‐dried, or herbarium tissue samples across many herbaceous and woody plant families, with yields and quality suitable for downstream PCR applications.

## AUTHOR CONTRIBUTIONS

F.E.G. analyzed data and wrote the manuscript. Y.H.G. designed and carried out experiments and analyzed data. M.R.P. analyzed data and wrote the manuscript. All authors gave their approval of the final manuscript before submission and publication.

## Data Availability

The data provided in this study are fully discoverable, freely reusable, and citable. The raw sequence data used for the development of *Sassafras albidum* primers were obtained from the 1KP Transcriptomes Consortium available in the National Center for Biotechnology Information Sequence Read Archive (SRA) database under BioProject accession PRJEB4922.

## APPENDIX 1 Herbarium taxa used in this study.

**Table:**

Accession no.^a^	Taxon	Collection date	Collector
NA 0095479	*Hemerocallis thunbergii* Baker	8 Aug. 1984	Yinger et al. 2196
NA 0098630	*Ilex opaca* Aiton	12 Oct. 2003	Henry 125
NA 0095480	*Mimulus aurantiacus* Curtis	11 June 2004	Povich s.n.
NA 0095477	*Onoclea sensibilis* L.	28 June 1995	Altvatter & Hammond 26‐95
NA 0057063	*Panicum virgatum* L.	1 Oct. 2008	Webster 3169
NA 0103576	*Pinus pinceana* Gordon	22 Aug. 1994	Silba B‐68
NA 0095478	*Salix caroliniana* Michx.	22 Apr. 1993	Godfrey 84535
NA 0034557	*Solanum oplocense* Hawkes	30 Aug. 1984	Spjut 8568

^a^ All specimens are from the Herbarium at the U.S. National Arboretum (NA).

## APPENDIX 2 Detailed protocol for the extraction of DNA from recalcitrant plant species.

### Reagents

- 100% acetone
- 1% SDS (w/v) in TE buffer
- TE buffer (10 mM Tris [pH 8.0], 1 mM EDTA)
- Nuclease‐free water
- 95% ethanol (for cleaning equipment)

### Supplies/equipment

- 1.5‐mL microcentrifuge tubes
- 5‐mm cork borer
- Forceps
- Metal rod (~2 mm diameter)
- Vortexer
- Microcentrifuge
- Dry block incubator
- Ice

### Extraction protocol

1. Add 0.5–1.0 mL of 100% acetone to a 1.5‐mL microcentrifuge tube (enough to immerse the leaf tissue prepared in Step 2). Caution: Acetone is toxic, so please follow all recommendations from the Material Safety Data Sheet (MSDS).
2. Cut up to three disk(s) 5 mm in diameter with a clean cork borer from a fresh, dried, or frozen leaf. Clean the cork borer with 95% ethanol between samples to prevent cross‐contamination.
3. Transfer the leaf disk(s) into the microcentrifuge tube containing acetone for fixation and leave in the acetone for at least 2 h at room temperature or until extraction. Change acetone solution one or two times until the acetone discoloration stops and the sample turns white.
4. Remove the fixed leaf disk(s) from the acetone tube using forceps. Properly discard the acetone solution and air‐dry the leaf disk(s) to allow the acetone to evaporate from the sample.
5. Place the air‐dried disk(s) into a new 1.5‐mL microcentrifuge tube and add 200 µL of 1% SDS.
6. Grind the leaf disk with the SDS in the microfuge tube into small pieces with a metal rod.
7. Vortex the tube and incubate at 90°C for 20 min in the dry block incubator, with occasional vortexing during the incubation.
8. Place the tube on ice for 5 min and vortex.
9. Centrifuge at 14,000 rpm for 5 min.
10. Transfer the supernatant to a new 1.5‐mL microcentrifuge tube and add 200 µL of 100% acetone and vortex.
11. Centrifuge at 6000 rpm for 1 min and discard the supernatant.
12. Resuspend the pellet in 100 µL of nuclease‐free water. The DNA can now be quantified and used for downstream PCR applications.
